# HBV infection increases the risk of macular degeneration: the roles of HBx-mediated sensitization of retinal pigment epithelial cells to UV and blue light irradiation

**DOI:** 10.1186/s12967-018-1594-4

**Published:** 2018-08-10

**Authors:** Ruey-Hwang Chou, Chang-Yin Lee, Lee-Won Chong, Kuang-Hsi Chang, Cheng-Li Lin, Ke-Sin Yan, Chingfu Tsou, Yi-Chao Hsu

**Affiliations:** 1 Graduate Institute of Biomedical Sciences and Center for Molecular Medicine, China Medical University, Taichung, Taiwan; 2 Department of Biotechnology, Asia University, Taichung, Taiwan; 3 College of Medicine, The School of Chinese Medicine for Post Baccalaureate, I-Shou University, Yancho Campus, Kaohsiung, Taiwan; 4 Department of Chinese Medicine, E-DA Hospital, Kaohsiung, Taiwan; 5 Department of Chinese Medicine, E-DA Cancer Hospital, Kaohsiung, Taiwan; 6 Division of Hepatology and Gastroenterology, Department of Internal Medicine, Shin Kong Wu Ho-Su Memorial Hospital, Taipei, Taiwan; 7 School of Medicine, Fu-Jen Catholic University, New Taipei City, Taiwan; 8 Department of Medical Research, Tungs’ Taichung Metroharbor Hospital, Taichung, Taiwan; 9 Graduate Institute of Biomedical Sciences, China Medical University, Taichung, Taiwan; 10 Management Office for Health Data, China Medical University Hospital, Taichung, Taiwan; 11 Department of Automatic Control Engineering, Feng Chia University, Taichung, Taiwan; 12 Institute of Biomedical Sciences, Mackay Medical College, No. 46, Sec. 3, Zhongzheng Rd, Sanzhi Dist, New Taipei City, 252 Taiwan

**Keywords:** Hepatitis B virus, HBx, Macular degeneration, ARPE19

## Abstract

**Background:**

Hepatitis B virus (HBV) infection is strongly associated with hepatocellular carcinoma due to the main pathogenic X protein of HBV (HBx). Whether HBV infection and the HBx protein could result in macular degeneration (MD) is not known. The aim of this study is to assess the association and underlying mechanisms between HBV infection and MD.

**Methods:**

The National Health Research Institutes in Taiwan built a large database, the National Health Insurance Research Database (NHIRD), which includes the claims data from the Taiwan National Health Insurance (NHI) program. The Taiwan NHI is a single-payer, compulsory health insurance program for Taiwan citizens. The data for the present study were derived from the Longitudinal Health Insurance Database, which contains the claims data of 1 million insured people within the NHIRD, including beneficiary registration, inpatient and outpatient files, drug use, and other medical services. In this study, we first investigated the association of HBV infection and the risk of MD by a population-based cohorts study enrolling 39,796 HBV-infected patients and 159,184 non-HBV-infected patients.

**Results:**

After adjustment of age, sex, and comorbidities, the risk of MD was significantly higher in the HBV-infected cohort than in the non-HBV-infected cohort (adjusted HR = 1.31; 95% CI = 1.17–1.46). In vitro, we provided evidence to demonstrate that overexpression of HBx in the human retinal pigment epithelial (RPE) cell line, ARPE19, significantly reduced cell viability and clonogenic survival upon UV and blue light irradiation. By gene microarray analysis, we further showed that almost all genes in DNA repair pathways including base excision repair, nucleotide excision repair, mismatch repair, and homologous recombination were significantly down-regulated in the UV-induced cell death of HBx-transfected ARPE19 cells.

**Conclusions:**

The HBx protein may sensitize RPE cells to UV and blue light irradiation and increase the risk of HBV-infection-associated MD through down-regulation of multiple DNA repair pathways.

**Electronic supplementary material:**

The online version of this article (10.1186/s12967-018-1594-4) contains supplementary material, which is available to authorized users.

## Background

Macular degeneration (MD) is a retinal disease and is the main cause of blindness in elderly [1]. The precise etiology of MD and age-related MD (AMD) is still unclear; however, the roles of sunlight ultraviolet (UV) exposure has been demonstrated in the pathogenesis of AMD [1]. UV irradiation and blue light irradiation both induce cellular damage through the generation of reactive oxygen species and oxidative stress, which have been considered the major pathological causes of photoreceptor cell death in AMD [2, 3]. However, the underlying etiopathogenesis mechanism is poorly understood. Therefore, investigating how risk factors initiate early retinal damage and developing therapeutic strategies for the prevention of its progression are necessary.

Hepatitis B virus (HBV) is a major etiology of hepatic malignancy, and it has a chronic disease course [4]. The HBV genome comprises four overlapping open reading frames (ORFs): C, P, S, and X. The X-ORF encodes the HBV X protein (HBx) with a 154-amino-acid-long peptide and a molecular mass of 17.5 kDa [5]. The HBx protein has been demonstrated to activate several signaling pathways such as the Ras and Raf MAPK signaling pathways in transformation [6] and proliferation [7] and the SAPK/JNK and PI3K-Akt-Bad signaling pathways in survival [8, 9]. Notably, HBx may activate or inhibit apoptotic pathways, depending on the scenario. For example, HBx may enhance apoptosis by interacting with c-FLIP and Bax [10, 11], transactivating the gene expression levels of Fas ligand [12], reducing mitochondrial membrane potential [13], and altering intracellular Ca^2+^ homeostasis [14] and Bcl2-mediated inhibitory effects [15]. By contrast, HBx can inhibit apoptosis by interacting with survivin-HBXIP [16] or inactivating p53 [17], caspase 3 [18], and Fas-mediated apoptosis [8].

The underlying mechanisms of how and why HBx enhances or inhibits apoptosis in different cellular contexts remain unclear. Research has shown that HBV can replicate in many extrahepatic cell types including neuronal cells, fibroblasts, keratinocytes, hematopoietic precursors, macrophages/monocytes, sustentacular cells, endothelial cells, and mucosal epithelial cells [19]. The presence of HBV in different tissues and cell types may account for the extrahepatic syndromes associated HBV infection, such as neuropathy, vasculitis, and dermatitis [19]; HBV has also been detected in the eye [20–22]. However, the pathogenic mechanisms of HBV-infection-associated extrahepatic syndromes must be explored and clarified. In the present study, we developed a hypothesis about the potential association between HBV infection and MD. Thus, we conducted a nationwide population-based cohort study and demonstrated that HBV infection increases the risk of subsequent MD. Here, we provide several lines of in vitro evidence to reveal that base excision repair (BER), nucleotide excision repair (NER), mismatch repair (MMR), homologous recombination (HR), DNA replication, the cell cycle, the p53 signaling pathway, cancer-related pathways, circadian rhythms, and the tumor necrosis factor (TNF) signaling pathway, are involved in the UV-induced cell death of HBx-expressing cells and HBV-infection-associated MD. Our findings may facilitate the development of preventive strategies toward these mechanisms for HBV-infection-associated MD.

## Patients, materials, and methods

### Data source

The National Health Research Institutes (NHRI) built a large database, the National Health Insurance Research Database (NHIRD), which includes the claims data from the Taiwan National Health Insurance (NHI) program. The Taiwan NHI is a single-payer, compulsory health insurance program for Taiwan citizens. The data for the present study were derived from the Longitudinal Health Insurance Database (LHID), which contains the claims data of 1 million insured people within the NHIRD, including beneficiary registration, inpatient and outpatient files, drug use, and other medical services. One million insured people were randomly selected between 1 January 1996, and 31 December 2000, and followed up in the LHID. Notably, the disease record system in the Taiwan NHI was established according to the International Classification of Diseases, Ninth Revision, Clinical Modification (ICD-9-CM). To protect the privacy of those insured, the NHRI replaced the original identification numbers with anonymous numbers before releasing the database publicly.

### Study population

To investigate the association between MD risk and HBV infection, we designed a retrospective population-based cohort study and established an HBV-infected cohort and a comparison cohort. The HBV-infected cohort comprised patients with newly onset HBV (ICD-9-CM 070.20, 070.22, 070.30, 070.32, and V02.61) who were aged ≥ 20 years; the patients were recruited from 1 January 2000, to 31 December 2011, and the index date was ordered as the day of the HBV diagnosis. The comparison cohort was formed by selecting individuals in the LHID without a history of HBV infection and frequency matching them against the patients at a ratio of 1:4 (HBV-infected cohort vs. comparison cohort); the matching criteria included age. The index dates of the comparisons were randomly assigned a month and a day, as well as the same index year as the matched cases. We excluded patients with a history of HCV infection (ICD-9-CM 070.41, 070.44, 070.51, 070.54, and V02.62) or MD (ICD-9-CM 362.5) before the index date. The observation outcome of interest was the occurrence of an MD event. We observed these two cohorts at the index date and stopped the follow-up process when the patients were removed from the Taiwan NHI, developed MD, or on December 31, 2011, whichever occurred first. Age, sex, and comorbidity are the common confounding factors in NHIRD research; we therefore collected the patients’ comorbidity histories prior to the index date. Specifically, the comorbidities examined in this study comprised hypertension (ICD-9-CM 401–405), hyperlipidemia (ICD-9-CM 272), alcohol-related illness (ICD-9-CM 291, 303, 305, 571.0, 571.1, 571.2, 571.3, 790.3, A215, and V11.3), diabetes (ICD-9-CM 250), asthma (ICD-9-CM 493), cirrhosis (ICD-9-CM 571.2, 571.5, and 571.6), anxiety (ICD-9-CM 300.00), and coronary artery disease (ICD-9-CM 410–414).

### Cell culture

ARPE19 cells, which form a human retinal pigment epithelial cell line (ATCC number: CRL-2302), were used in this study. The cells were cultured in Dulbecco’s Modified Eagle’s Medium (DMEM)/Nutrient Mixture F-12 medium (Hyclone), supplemented with 10% fetal bovine serum (Hyclone), 100 U/mL penicillin, and 100 µg/mL streptomycin, in a humidified incubator at 37 °C and with 5% CO_2_. The cells were subcultured every 2–3 days to maintain exponential growth.

### Light sources

The UV crosslinker, CL-1000L model (Ultra-Violet Products, LCC, CA, USA), was used as the source of UV light. The UV light source consists of five tubes of 8 W UV dual bipin discharge type (115 V/60 Hz/0.7 A), and the wavelength of the UV tube is 365 nm (UV-A). Two operational settings are available in the UV crosslinker: (1) preset UV energy exposure and (2) preset UV time exposure. The energy needed in the experiments can be set on the touch pad. It takes 4 min for the UV light exposure to 1 J/cm^2^. The exposure time of the UV light depends on the energy used in our experiments. The blue light LED lamp, MIC-209 model (60 W, blue light), was used as the source of visible blue light. The wavelength and the chromaticity diagram were measured by using the spectrometer USB2000+ (Ocean Optics, FL, USA) and the luminance colorimeter BM-7A (Topcon Tech. Co., Tokyo, Japan), respectively. The irradiance of the blue LED lamp was directly measured by a solar power meter SPM1116SD (Lutron Electronic Enterprise Co., Ltd., Taiwan) at the distance of 6.5 cm below the blue light source, where the cultured cells were exposed in the experiments.

### Cell viability assay

Cell viability was analyzed and measured using the 3-[4,5-dimethylthiazol-2-yl]-2,5 diphenyltetrazolium bromide (MTT) method. Briefly, the cells were seeded in a 35-mm dish at a density of 2 × 10^5^ cells/cm^2^. After overnight culturing, the cultured medium was discarded and the cells were exposed to the indicated dose of UV radiation, followed by a replacement of fresh medium at 37 °C. After incubation for a different period, MTT (Sigma, St. Louis, MO, USA) was added to a final concentration of 0.5 mg/mL and incubated in a CO_2_ incubator for an additional 4 h. Subsequently, the medium was aspirated, and 500 μL of dimethyl sulfoxide (Sigma, St. Louis, MO, USA) was added to the dish to dissolve formazan crystals. The absorbance was then obtained using a Synergy 2 microplate reader (BioTek Instruments, VT, USA) at a test wavelength of 490 nm with a reference wavelength of 630 nm. Finally, cell viability was determined by the relative absorbance of the experimental treatment compared with that of the control treatment.

### DNA transfections

HBx plasmids were propagated in *Escherichia coli* and isolated with a Midi plasmid kit (Geneaid). Transfection of the ARPE19 cells was then achieved by using the TransIT-X2 reagent (Mirus) according to the user manual. In brief, approximately 80% of confluent cells were used for transfection, with 7.5 μL of TransIT-X2 and 2.5 μg of plasmid DNA in a 6-well plate format. After 24 h, the transfected cells were subcultured and a stable transfectant was generated by adding G418 (Enzo) at a final concentration of 0.5 mg/mL.

### Colony formation assay

Two thousand cells were seeded into a 60-mm dish. After 24 h, the cells were exposed to the indicated dose of UV irradiation and cultured with fresh medium for 2 weeks. Subsequently, the cells were fixed with a 4% paraformaldehyde solution and stained with 0.1% crystal violet for 30 min. After washing, the crystal violet was dissolved with 10% acetic acid and the absorbance was measured at 590 nm. The relative colony number was calculated according to the relative absorbance of the experimental treatment in comparison with that of the control treatment.

### Human oligonucleotide DNA microarray

Following treatment, the total RNAs of each group of cells were extracted using the Trizol reagent (Invitrogen, Carlsbad, CA, USA). The RNA yields and purity were checked by OD260/OD280 (> 1.8) and OD260/OD230 (> 1.6) using an Agilent 2100 Bioanalyzer (Agilent Technologies, Santa Clara, CA, USA). Additionally, we used the human oligonucleotide DNA microarray (Human Whole Genome OneArray^®^v6, Phalanx Biotech Group, Taiwan), which contains 32,679 DNA oligonucleotide probes. Of these probes, 31,741 correspond to the annotated genes in the RefSeq v51 and Ensembl v65 databases. To control the experiment quality, the remaining 938 control probes were also included. Detailed descriptions of the gene array list are available from http://www.phalanx.com.tw/Products/HOA_Probe.php.

### Data analysis and clustering

For the in vitro studies, the experiments were performed, at a minimum, in triplicate. In each experiment, the mean value of the repetitions was calculated and then used in the statistical analysis. All of the data were normalized to control the values of each assay and are presented as the mean ± SD. Additionally, the data were analyzed using one-way ANOVA, and significance was again set at *P* < 0.05. The array data were analyzed using the Rosetta Resolver System (Rosetta Biosoftware), which was applied to correct the data by removing both systematic and random errors. Signals that passed the criteria were then normalized through a 50% median scaling normalization method. The technical repeat data were analyzed by calculating the Pearson correlation coefficient to review the reproducibility (R value ≥ 0.975), and then the normalized data were transformed to gene expression log2 ratios between the mock and HBx groups. Signals with a log2 ratio of ≥ 1 or log2 ratio of ≤ −1 and a *P* value of < 0.05 were selected and defined as differentially expressed (DE) genes for further analysis. Scatter plots were made to visually assess the variation between chips. In addition, volcano plots (Fig. 4a) and hierarchical clustering (Fig. 4c) were performed to visually demonstrate distinguishable gene expression profiles among samples.

### Statistical analyses for population-based study

We measured the age distributions of the two cohorts by mean and standard deviation (SD), and examined the sex and comorbidity distributions by number and percentage. The age distribution differences were tested using two-sample t-tests, whereas the sex and comorbidity distribution differences were assessed using a Chi square test. We applied two strategies to investigate the risk of MD between the HBV-infected and comparison cohorts. First, the incidence density of developing MD for the two cohorts was calculated, and the cumulative incidence curves were evaluated using the Kaplan–Meier method; specifically, the log-rank test was applied to assess the incidence curve differences between the HBV-infected and comparison cohorts. Second, the crude and adjusted hazard ratios (aHRs) and 95% confidence intervals (CIs) were estimated using Cox proportional hazard models; specifically, a stratified analysis was used to demonstrate the risk of MD in the HBV-infected cohort compared with the comparison cohort, according to age, sex, and comorbidity. All statistical analyses were conducted using SAS 9.4 software (SAS Institute, Cary, NC, USA), and the cumulative curve was plotted using R software (R Foundation for Statistical Computing, Vienna, Austria). Significance was set at *P* < 0.05 for two-sided testing.

## Results

### HBV infection may increase the risk of MD

From the LHID, 39,796 HBV-infected patients, along with 4-fold comparison patients, were enrolled in this study (Table 1). Their mean age was nearly 44 years and 58% were male. The most common comorbidities in the HBV-infected cohort were hypertension (21.7%), hyperlipidemia (19.0%), and coronary artery disease (10.2%); moreover, the percentages of all comorbidities were significantly greater in the HBV-infected cohort than in the comparison cohort (all *P *< 0.001).

**Table 1:** Demographic characteristics and comorbidities in cohorts with and without HBV infection

Variable	HBV infection		*P* value
	No	Yes	
	N = 159,184	N = 39,796	
*Age, years*			0.99
≤ 34	48,116 (30.2)	12,029 (30.2)	
35–49	59,192 (37.2)	14,798 (37.2)	
50+	51,876 (32.6)	12,969 (32.6)	
Mean ± SD^†^	44.0 ± 14.8	44.2 ± 14.4	0.01
*Sex*			0.99
Female	66,696 (41.9)	16,674 (41.9)	
Male	92,488 (58.1)	23,122 (58.1)	
*Comorbidity*			
Cirrhosis	547 (0.34)	2305 (5.79)	< 0.001
Diabetes	8391 (5.27)	3140 (7.89)	< 0.001
Hypertension	29,134 (18.3)	8651 (21.7)	< 0.001
Hyperlipidemia	20,199 (12.7)	7556 (19.0)	< 0.001
Asthma	6779 (4.26)	2252 (5.66)	< 0.001
Coronary artery disease	12,879 (8.09)	4068 (10.2)	< 0.001
Alcohol-related illness	4669 (2.93)	2539 (6.38)	< 0.001
Anxiety	7039 (4.42)	2911 (7.31)	< 0.001

The numbers in parentheses are the percent of the total group number. Chi Square Test; ^†^ T-Test

**Fig. 1:**
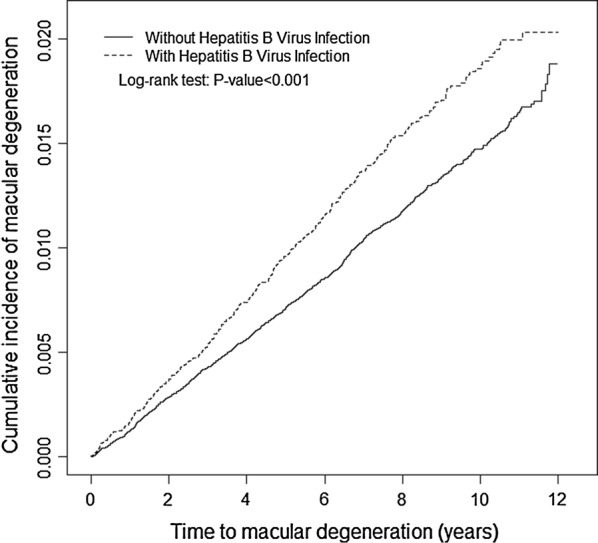
Association of HBV infection and MD. During a 12-year follow-up, we accumulated 450 and 1441 cases of MD from 39,796 HBV-infected patients and 159,184 non-HBV-infected patients, respectively. The cumulative incidence of MD in the patients with (dashed line) or without (solid line) HBV infection is shown

MD occurred in 450 and 1441 patients in the HBV-infected and comparison cohorts, respectively (Table 2). Notably, the incidence of MD was nearly 1.29-fold higher among the HBV-infected patients than among the non-HBV-infected patients (1.90 vs. 1.47/1000 person-years). Figure 1 illustrates the incidence curve of MD (log-rank test *P* < 0.001). After adjustment for age, sex, and the comorbidities, the HBV-infected patients had a 1.31-fold increased risk of developing MD compared with the non-HBV-infected patients (HR = 1.31, 95% CI = 1.17–1.46).

**Table 2:** Incidence and HR for MD and associated risk factors

Variable	Event	Person years (PY)	Rate^#^	Crude HR (95% CI)	Adjusted HR^†^ (95% CI)
HBV infection					
No	1441	977,388	1.47	1.00	1.00
Yes	450	237,163	1.90	1.29 (1.16, 1.43)***	1.31 (1.17, 1.46)***
Age, years					
≤ 34	81	407,821	0.2	1.00	1.00
35–49	294	458,479	0.64	3.26 (2.55, 4.17)***	2.93 (2.29, 3.75)***
50+	1516	348,251	4.35	22.4 (17.9, 28.0)***	14.4 (11.4, 18.2)***
Sex					
Female	857	510,692	1.68	1.14 (1.04, 1.25)**	0.96 (0.88, 1.05)
Male	1034	703,859	1.47	1.00	1.00
Comorbidity					
Cirrhosis					
No	1853	1,203,531	1.54	1.00	1.00
Yes	38	11,020	3.45	2.26 (1.64, 3.12)***	0.99 (0.71, 1.37)
Diabetes					
No	1552	1,158,117	1.34	1.00	1.00
Yes	339	56,434	6.01	4.54 (4.03, 5.11)***	1.51 (1.33, 1.71)***
Hypertension					
No	937	1,010,834	0.93	1.00	1.00
Yes	954	203,717	4.68	5.10 (4.66, 5.59)***	1.44 (1.29, 1.61)***
Hyperlipidemia					
No	1228	1,062,951	1.16	1.00	1.00
Yes	663	151,600	4.37	3.82 (3.47, 4.19)***	1.28 (1.15, 1.54)***
Asthma					
No	1699	1,168,449	1.45	1.00	1.00
Yes	192	46,102	4.16	2.89 (2.41, 3.35)***	1.33 (1.14, 1.54)***
Coronary artery disease					
No	1353	1,124,263	1.20	1.00	1.00
Yes	538	90,288	5.96	4.99 (4.52, 5.52)***	1.41 (1.26, 1.58)***
Alcohol-related illness					
No	1831	1,181,964	1.55	1.00	1.00
Yes	60	32,587	1.84	1.20 (0.93, 1.55)	–
Anxiety					
No	1691	1,166,7	1.45	1.00	1.00
Yes	200	47,845	4.18	2.92 (2.52, 3.39)***	1.34 (1.15, 1.56)***

*HR* hazard ratios, *CI* confidence intervals

Rate#: incidence rate per 1000 person-years; Crude HR*: relative hazard ratio; Adjusted HR†: multivariable analysis, including age, sex, and the comorbidities of cirrhosis, diabetes, hypertension, hyperlipidemia, asthma, coronary artery disease, and anxiety

* *P *< 0.05, ** *P *< 0.01, *** *P *< 0.001

Table 3 presents the risk of MD development in the HBV-infected patients compared with the non-HBV-infected patients, according to age, sex, and the presence of certain comorbidities. The risk of MD was nearly 1.24-fold higher in the HBV-infected cohort than that in the comparison cohort for the patients aged ≥ 50 years (HR = 1.24, 95% CI = 1.10–1.40); by contrast, there was no statistical significance regarding the risk of MD between the HBV-infected cohort and the comparison cohort among patients aged ≤ 35 (HR = 0.99, 95% CI = 0.58–1.71), nor in those aged 35–49 years (HR = 1.28, 95% CI = 0.98–1.71). Additionally, the risks of developing MD were both nearly 1.3-fold greater in the male HBV-infected patients (HR = 1.30, 95% CI = 1.12–1.51) and female HBV-infected patients (HR = 1.31, 95% CI = 1.12–1.54), compared with the comparison cohort. Among the entire study population, the HRs of developing MD were 1.47 (95% CI = 1.20–1.80) and 1.23 (95% CI = 1.08–1.39) when there was no comorbidity and at least one comorbidity, respectively.

**Table 3:** Incidence of MD by age, sex, and comorbidity, and Cox-model-measured HRs for patients with and without HBV infection

Variables	HBV infection						Crude HR^*^ (95% CI)	Adjusted HR^†^ (95% CI)
	No			Yes				
	Event	PY	Rate^#^	Event	PY	Rate^#^		
*Age, years*								
≤ 34	64	325,136	0.20	17	82,685	0.21	1.04 (0.61, 1.78)	0.99 (0.58, 1.71)
35–49	218	368,374	0.59	76	90,104	0.84	1.42 (1.10, 1.85)**	1.28 (0.98, 1.67)
50+	1159	283,877	4.08	357	64,374	5.55	1.37 (1.21, 1.54)***	1.24 (1.10, 1.40)***
*Sex*								
Female	651	409,645	1.59	206	101,047	2.04	1.28 (1.10, 1.50)**	1.31 (1.12, 1.54)***
Male	790	567,743	1.39	244	136,116	1.79	1.29 (1.12, 1.49)***	1.30 (1.12, 1.51)***
*Comorbidity*								
No	488	714,950	0.68	114	143,537	0.79	1.16 (0.95, 1.42)	1.47 (1.20, 1.80)***
Yes	953	262,438	3.63	336	93,626	3.59	0.99 (0.87,1.12)	1.23 (1.08, 1.39)**

*HR* hazard ratios, *CI* confidence intervals

Rate^#^: incidence rate per 1000 person-years; Crude HR*: relative hazard ratio; Adjusted HR^†^: multivariable analysis, including age, and the comorbidities of cirrhosis, diabetes, hypertension, hyperlipidemia, asthma, coronary artery disease, and anxiety

* *P *< 0.05, ** *P *< 0.01, *** *P *< 0.001

### HBx expression did not affect cell morphology or cell growth of the ARPE19 cells

**Fig. 2:**
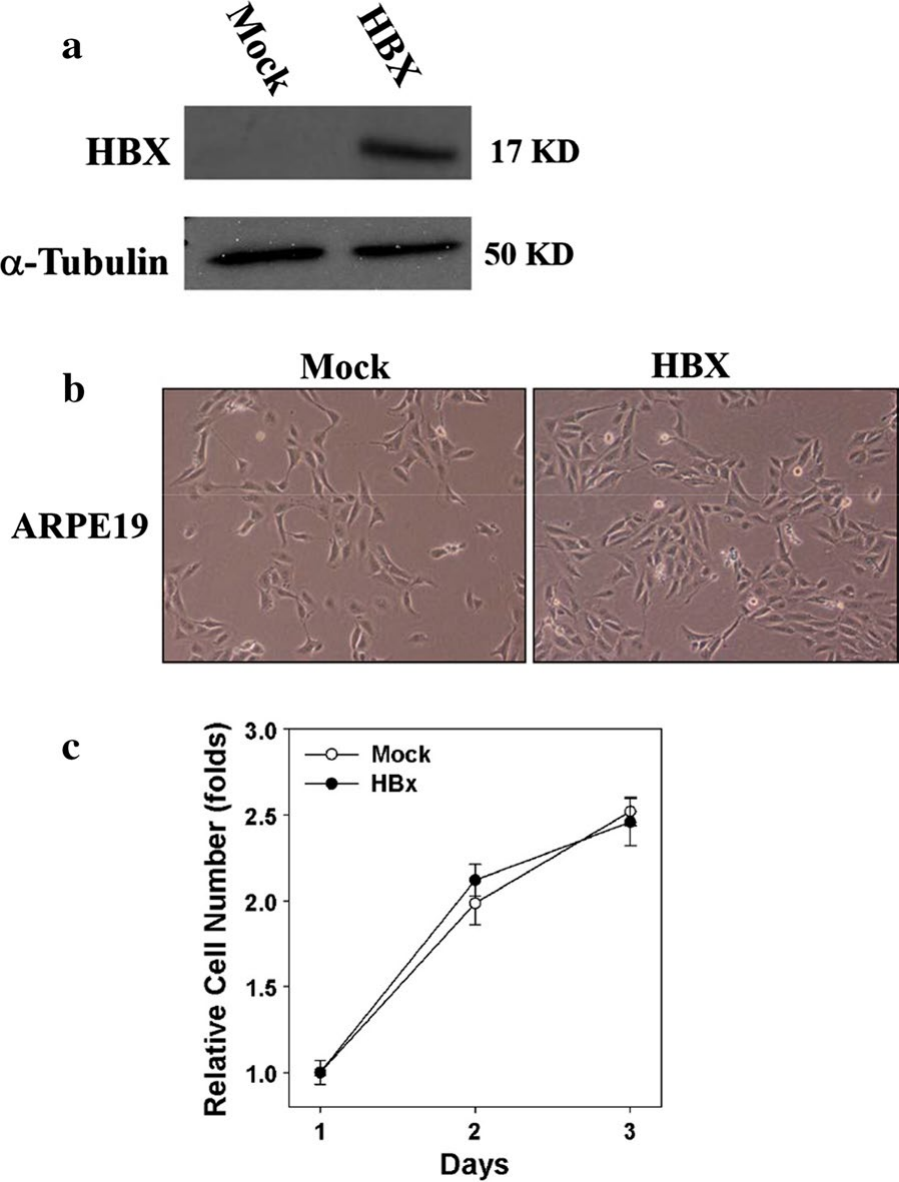
Effects of the HBx protein on morphology and growth rate in retinal epithelial cells. ARPE19 cells were transfected with a mock plasmid or HBx-containing plasmid, followed by a selection with 0.5 mg/mL G418 for several weeks to establish stable transfectants. **a** Whole-cell lysate was extracted from each transfectant, and the expression of the HBx protein was examined using Western blotting. **b** Morphology of each stable transfectant was observed under a phase-contrast microscope at ×400 magnification. **c** Cell growth rate of each transfectant was measured using MTT assay

To determine the functional effects of the HBx protein in retinal epithelial cells, ARPE19 cells were stably transfected with either a mock plasmid or HBx-containing plasmid. As depicted in Fig. 2a, the HBx protein was stably expressed in the HBx-transfected ARPE19 cells, after being cultured in 0.5 mg/mL G418 for several weeks. Previous studies have reported that the expression of HBx induces spontaneous apoptotic cell death in mouse fibroblasts [23], human HepG2 cells [24, 25], and the liver of HBx transgenic mice [26]. However, we found that the expression of HBx at tolerant levels was not deleterious toward ARPE19 cells. In addition, the morphology (observed under a phase-contrast microscope; Fig. 2b) and cell growth rate (measured by the MTT assay; Fig. 2c) of the HBx- and mock-transfected cells were similar.

### HBx protein sensitizes ARPE19 cells to UV-induced cell death

**Fig. 3:**
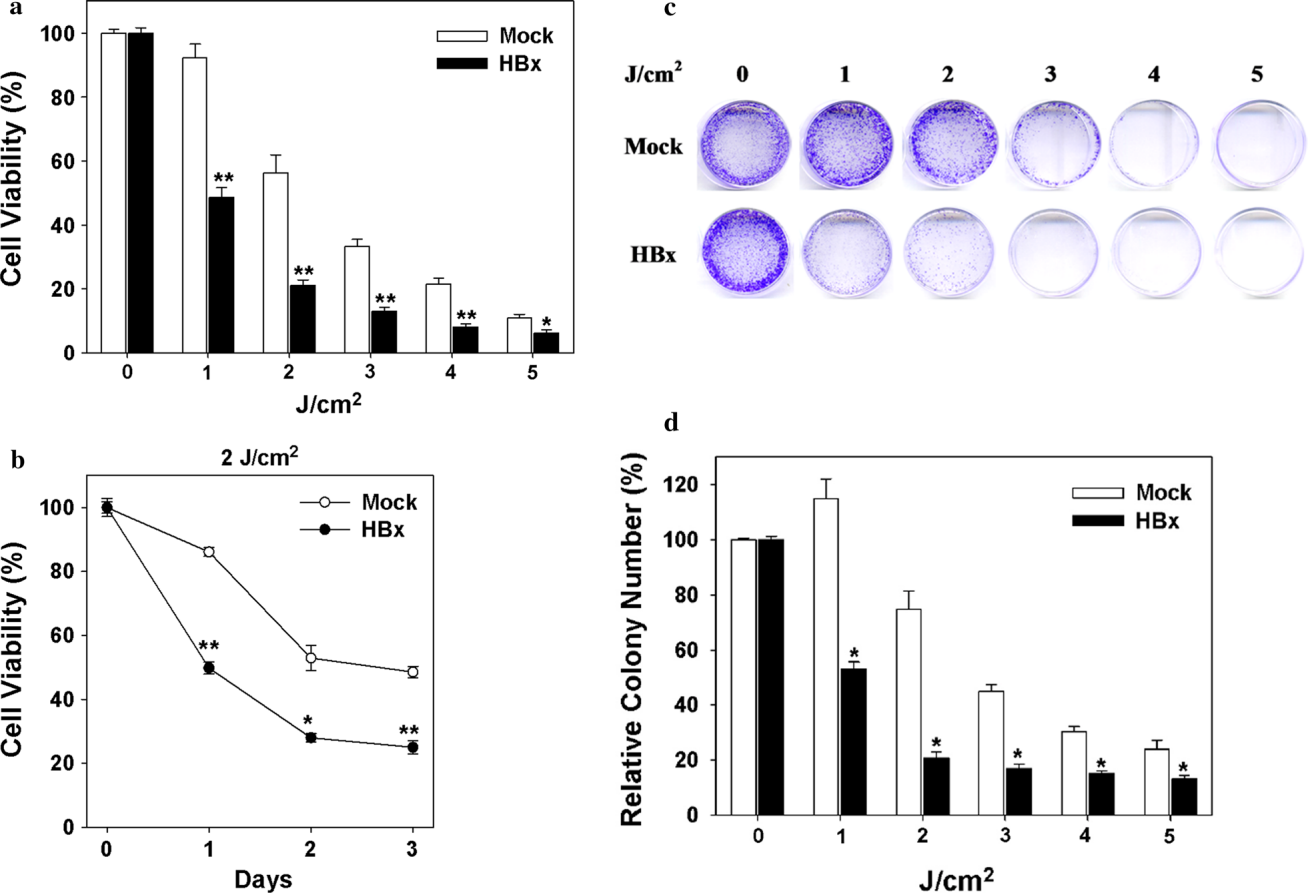
Cytotoxic effects of the HBx protein in response to UV irradiation in retinal epithelial cells. The cytotoxic effects, cell viability, and clonogenic survival were determined by the MTT assay and colony formation assay, respectively. **a** ARPE19 stable transfectants with or without (mock) HBx were exposed to the indicated dose of UV irradiation (0, 1, 2, 3, 4, and 5 J/cm^2^). Cell viability was examined through the MTT assay at 72 h post-irradiation. **b** ARPE19 stable transfectants were exposed to 2 J/cm^2^ UV, and cell viability was determined at the indicated time intervals (24, 48, and 72 h). **c**, **d** Cells were exposed to increased doses of UV irradiation, and clonogenic survival was determined through the colony formation assay. The representative result is depicted in (**c**), and the quantitative result from three independent experiments is shown in (**d**). Data are presented as mean ± SD. The symbols *P < 0.05 and **P < 0.01, respectively, compared with the control mock-transfectant

To clarify the cytotoxic effects of the HBx protein in retinal epithelial cells following UV irradiation, cell viability and clonogenic survival were measured using the MTT assay and colony formation assay, respectively. The results revealed that the expression of the HBx protein significantly decreased the cell viability of ARPE19 cells in dose- (from 1 to 5 J/cm^2^) and time- (from 1 to 3 days) dependent manner (Fig. 3). Similarly, the expression of the HBx protein markedly reduced the clonogenic survival of the ARPE19 cells in response to 1–5 J/cm^2^ UV irradiation (Fig. 3c, d).

### DE genes in HBx- and mock-transfected ARPE19 cells, pre- and post-UV irradiation

**Fig. 4:**
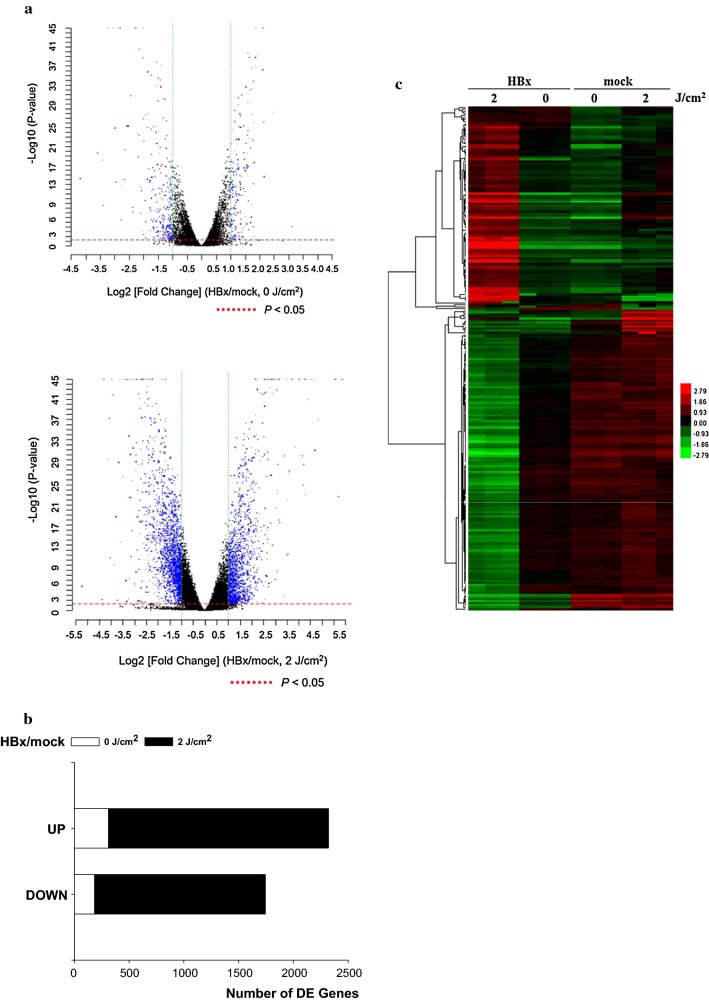
Transcriptional dialogue between HBx-transfected ARPE19-(HBx) and mock-transfected ARPE (mock) cells with and without UV irradiation. **a** Volcano plots of the sample with and without UV irradiation (mock vs. HBx). Standard selection criteria for identifying DE genes were established at log2 |fold change| ≧ 1 or ≤ − 1 and *P* < 0.05. **b** Number of upregulated or downregulated genes, compared with the expression levels, between mock-transfected and HBx-transfected cells with and without UVA/B irradiation (2 J/cm^2^). **c** Hierarchical clustering analysis of DE genes in mock-transfected cells versus HBx-transfected cells with and without UVA/B irradiation (2 J/cm^2^). The significantly DE genes following HBx infection (*P *< 0.05) from (**b**) were pooled and used to create heat maps. Genes are ordered in rows, and conditions are presented in columns. The red color indicates genes induced with UV irradiation versus without UV irradiation (fold change), and the green color denotes repressed genes

The global gene expression profiles of ARPE19 cells with or without the HBx protein were examined pre- and at 4 h post-UV irradiation. Standard selection criteria with log2 ratios of ≥ 1 (upregulated at least 2-fold) or log2 ratios of ≤ − 1.0 (downregulated at least 2-fold) and *P* < 0.05 were used to identify DE genes (Fig. 4a). Comparing the gene expression profiles from the HBx- and mock-transfected ARPE19 cells revealed that a total of 497 genes (185 upregulated and 312 downregulated) and 3569 genes (1560 upregulated and 2009 downregulated), respectively, were significantly DE pre- and post-UV irradiation (Fig. 4b). Moreover, these DE genes were hierarchically clustered, demonstrating a clear pattern of differential transcriptional regulation in cells with and without HBx pre- and post-UV irradiation (Fig. 4c).

### Functional classes of DE genes and their interaction networks in HBx- and mock-transfected ARPE19 cells, pre- and post-UV irradiation

**Fig. 5:**
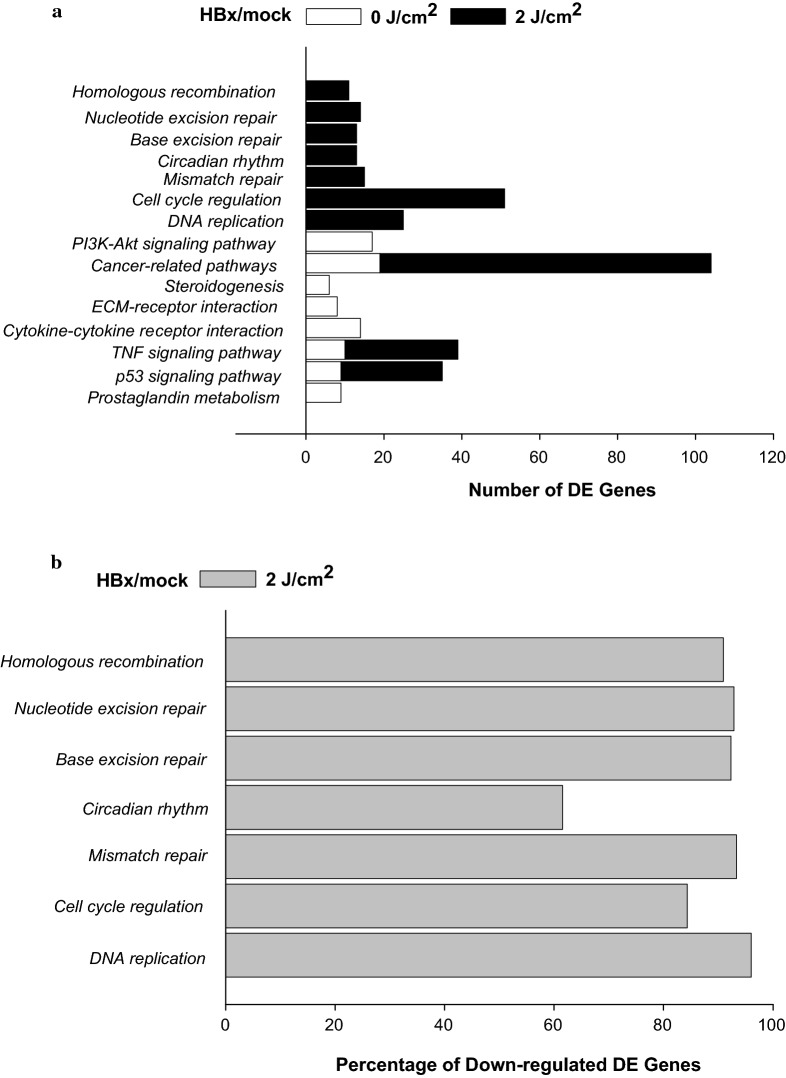
Functional annotation of genes in pathways in HBx-transfected ARPE19 (HBx) and mock-transfected ARPE19 (mock) cells. **a** The bar plot illustrates the DE genes involved in the indicated signaling pathways of HBx-transfected cells, compared with those of the mock-transfected cells pre- and post-UV-A/B irradiation (2 J/cm^2^). All DE genes are annotated using a generic GO-slim for biological processing. **b** The percentage of down-regulated DE genes in the particular pathways after UV irradiation was represented as the bar plot

To further functionally classify the DE genes in the HBx- and mock-transfected ARPE19 cells, an enrichment analysis was performed according to the multiple cellular process categories of the GO database. The results from the canonical pathway analysis of the HBx-transfected cells, compared with those of the mock-transfected cells, indicated that a significant number of the DE transcripts was assigned to functional categories of metabolic processes prior to UV irradiation: prostaglandin metabolism, the p53 signaling pathway, TNF signaling pathway, cytokine–cytokine receptor interaction, extracellular matrix–receptor interaction, steroidogenesis, various cancer-related pathways, and the PI3K-Akt signaling pathway. At 4 h post-UV irradiation, a significant number of the DE transcripts was reclassified into ten functional categories, namely BER, NER, MMR, HR, DNA replication, cell cycle regulation, p53 signaling pathway, various cancer-related pathways, circadian rhythms, and the TNF signaling pathway (Table 4 and Fig. 5a), in which most of them were down-regulated (Fig. 5b). Furthermore, according to the GO enrichment analysis, these DE genes were categorized into three groups predominantly involved in molecular functions (Table 5A), biological processes (Table 5B), and cellular components (Table 5C).

**Table 4:** Canonical pathway analysis for differentially expressed (DE) genes involved in ARPE19-HBx and ARPE19-control cells, pre- and post-UV irradiation

Pathway name	Genes in overlap (k)	*P* value
*HBx vs. mock*	*Before UV irradiation*	
1. Prostaglandin metabolism	9	1.52E−04
2. p53 signaling pathway	9	2.63E−04
3. TNF signaling pathway	10	1.39E−03
4. Cytokine-cytokine receptor	14	5.43E−03
5. ECM-receptor interaction	8	6.31E−03
6. Steroidogenesis	6	7.62E−03
7. Cancer-related pathways	19	1.03E−02
8. PI3K-Akt signaling pathway	17	1.37E−02
*HBx vs. mock*	*Post UV irradiation*	
1. DNA replication	25	7.62E−12
2. Cell cycle	51	2.57E−11
3. Mismatch repair	15	1.05E−06
4. p53 signaling pathway	26	1.31E−05
5. Cancer-related pathways	85	1.36E−03
6. Circadian rhythm	13	1.64E−03
7. TNF signaling pathway	29	3.03E−03
8. Base excision repair	13	3.05E−03
9. Nucleotide excision repair	14	2.57E−02
10 Homologous recombination	11	1.01E−02

**Table 5:** GO enrichment analysis of (A) molecular functioning, (B) biological processes, and (C) cellular components for the DE genes involved in ARPE19-HBx and ARPE19-control cells, pre- and post-UV irradiation

Gene set name	Genes in overlap	*P* value
*(A) Molecular functions*	*Before UV irradiation*	
1. Testosterone dehydrogenase (NAD +) activity	4	3.24E−04
2. Integrin binding	9	1.93E−03
3. Extracellular matrix binding	5	2.17E−03
4. Chemokine activity	6	3.93E−03
5. Cytokine activity	11	4.86E−03
6. Retinol dehydrogenase activity	4	6.34E−03
7. Heparan sulfate proteoglycan binding	4	6.34E−03
8. Heparin binding	10	8.49E−03
9. Chemoattractant activity	4	1.97E−02
10. MAP kinase tyrosine/serine/threonine phosphatase activity	3	3.08E−02
*Molecular functions*	*Post UV irradiation*	
1. Protein binding	1142	5.27E−20
2. Chromatin binding	94	6.79E−06
3. Single-stranded DNA-dependent ATPase activity	9	9.70E−06
4. Single-stranded DNA binding	30	6.45E−05
5. DNA binding	311	2.13E−04
6. Transcription factor binding	67	2.25E−04
7. DNA helicase activity	12	3.65E−04
8. Double-stranded DNA binding	26	6.50E−04
9. Insulin-like growth factor I binding	8	7.38E−04
10. Transcriptional activator activity, RNA polymerase II core promoter proximal region sequence-specific binding	55	8.53E−04
*(B) Biological processes*	*Before UV irradiation*	
Positive regulation of smooth muscle cell proliferation	11	2.03E−06
Cellular response to tumor necrosis factor	13	5.47E−06
Cellular response to interleukin-1	11	1.14E−05
Inflammatory response	24	1.23E−05
Positive regulation of p38MAPK cascade	6	1.72E−05
Cyclooxygenase pathway	5	6.50E−05
Positive regulation of endothelial cell proliferation	9	1.52E−04
Prostaglandin biosynthetic process	5	1.87E−04
Cell adhesion	24	2.02E−04
Negative regulation of cell proliferation	22	2.67E−04
*Biological processes*	*Post UV irradiation*	
DNA replication	77	1.27E−22
Cell division	114	2.75E−16
Mitotic nuclear division	84	8.94E−13
G1/S transition of mitotic cell cycle	44	5.69E−11
DNA replication initiation	22	1.55E−10
DNA repair	71	1.03E−08
Sister chromatid cohesion	39	4.31E−08
DNA strand elongation involved in DNA replication	13	4.52E−08
DNA synthesis involved in DNA repair	20	9.88E−08
Telomere maintenance via recombination	19	1.04E−07
*(C) Cellular components*	*Before UV irradiation*	
Extracellular region	66	6.60E−07
Extracellular space	53	4.71E−05
Proteinaceous extracellular matrix	16	8.91E−04
Endoplasmic reticulum	33	1.27E−03
Integral component of plasma membrane	49	1.39E−03
Synaptic vesicle	9	1.41E−03
Extracellular matrix	15	6.08E−03
Plasma membrane	110	9.46E−03
Organelle membrane	7	1.04E−02
Anchored component of membrane	8	1.11E−02
*Cellular components*	*Post-UV irradiation*	
Nucleoplasm	580	7.09E−20
Cytoplasm	941	1.79E−12
Nucleus	957	1.10E−10
Nuclear matrix	35	1.30E−06
Cytosol	598	2.40E−06
Chromatin	32	7.57E−06
Centrosome	99	8.62E−06
Nuclear chromosome, telomeric region	40	9.80E−06
Nuclear envelope	45	3.66E−05
Replication fork	11	3.98E−05

### HBx protein sensitizes ARPE19 cells to blue light-induced cell death

**Fig. 6:**
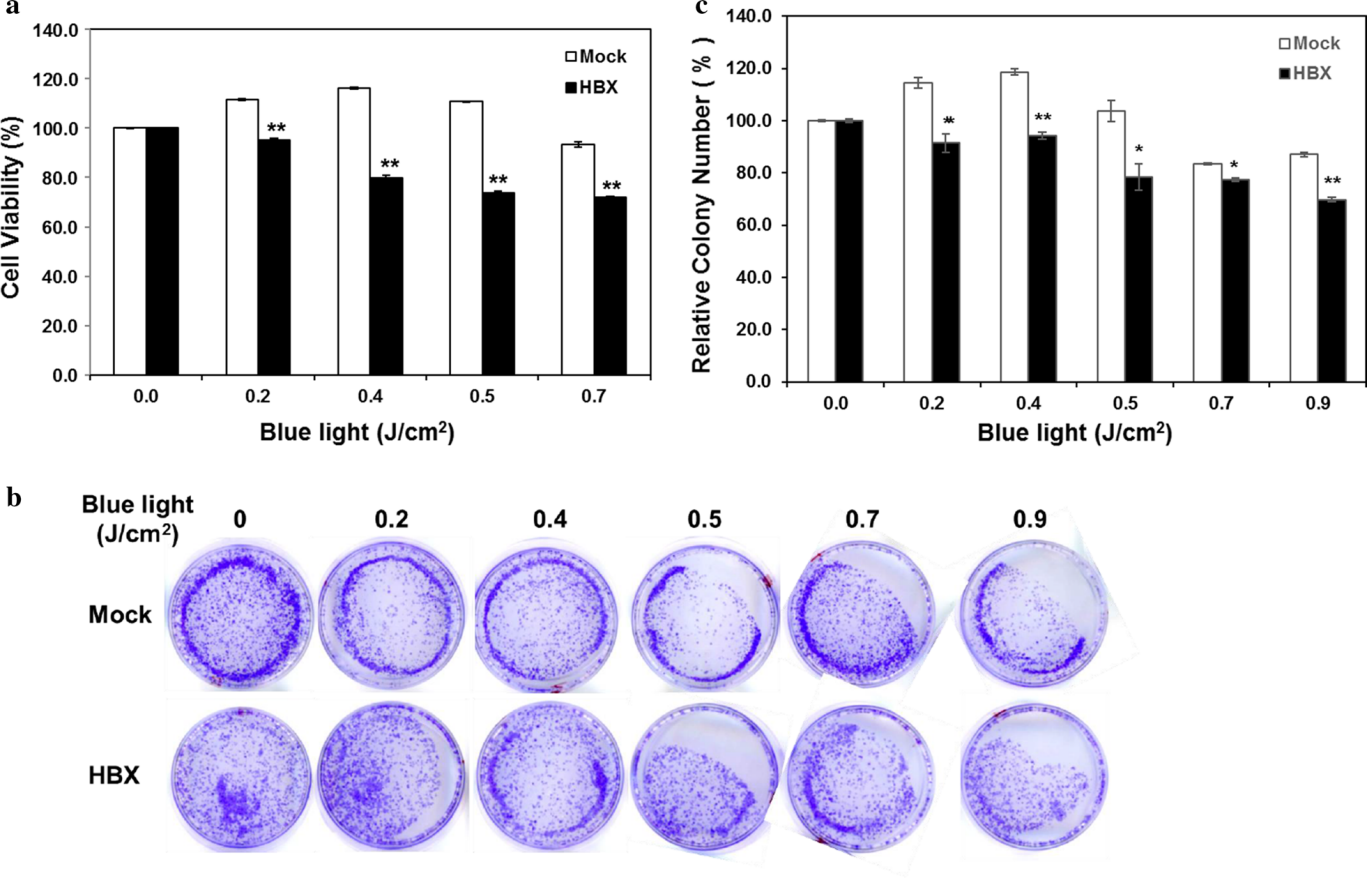
Cytotoxic effects of the HBx protein in response to blue light exposure in retinal epithelial cells. The cell viability and clonogenic survival were determined by the MTT assay and colony formation assay, respectively. **a** ARPE19 stable transfectants with or without HBx were exposed to the indicated dose of blue light. Cell viability was examined after 72 h. **b**, **c** Cells were exposed to increased doses of blue light, and clonogenic survival was determined after 2 weeks. The representative result is shown in (**b**), and the quantitative result from three independent experiments is shown in (**c**). Data are presented as mean ± SD. The symbols *P < 0.05 and **P < 0.01, respectively, compared with the mock-transfectant

To further clarify the effects of HBV infection on the sensitivity of retinal epithelial cells to short-wavelength visible light, the cytotoxic effects of the HBx protein in ARPE19 cells following blue light exposure were also determined. The wavelength of the blue light LED lamp peaked around 470 nm (Additional file 1: Figure S1A), the chromaticity diagram indicated the light belonged to blue light area (Additional file 1: Figure S1B), and the irradiance of the blue light was approximate 26 W/m^2^ at the distance of 6.5 cm below the light source, where the cells were exposed (Additional file 1: Figure S1C). The results of cell viability and clonogenic survival revealed that HBx-transfected ARPE19 cells were more sensitive to blue light than the mock-transfected ARPE19 cells in a dose-dependent manner (Fig. 6). In short, the HBx protein can sensitize retinal epithelial cells to UV irradiation and blue light exposure, suggesting that the HBx protein of HBV may be (at least partially) the critical factor that increases the incidence of MD in HBV-infected patients.

## Discussion

This is the first study to investigate the association and underlying mechanisms between chronic HBV infection and MD. We conducted a nationwide, population-based cohort study in Taiwan, with a matched comparison cohort, over a 12-year period; in addition, we used HBx-transfected human ARPE19 cells as the in vitro model. The major finding of our study is the significantly higher incidence of MD among patients with HBV infection. Furthermore, the patients with HBV infection exhibited a higher prevalence of cirrhosis, diabetes, hypertension, hyperlipidemia, asthma, coronary artery disease, alcohol-related illnesses, and anxiety than did the patients without HBV infection (all *P* < 0.001) (Table 1). Notably, the mean age of the HBV-infected patients was approximately 44 years, with the incidence of AMD significantly increasing among 35–49-year-olds (aHR = 2.93; 95% CI = 2.29–3.75) and those older than 50 (aHR = 14.4; 95% CI = 11.4–18.2). Because the onset of MD usually occurs in adolescence [27], our finding confirms that HBV-associated MD may be also age-dependent.

The incidence of MD was determined to increase with the presence of various comorbidities such as diabetes, hypertension, hyperlipidemia, asthma, coronary artery disease, or anxiety (Table 2). In previous studies, hypertension, hyperlipidemia, and coronary artery disease have also been suggested as risk factors for AMD [1, 28]. The integrity of highly polarized RPE cells is critical for maintaining retinal function because they are the major cell type responsible for AMD, with limited proliferative potential. The main characteristic of AMD is the death of RPE cells; thus, the human RPE cell line, ARPE19, has been utilized a cellular model to study the cellular and molecular mechanisms of AMD. Notably, ARPE19 cells have the characteristics of polarization and tight junction [29]. Previous studies have also suggested that the upregulation of ERK1/2 protects ARPE19 cells from oxidative-stress-induced cell death [30], whereas the inhibition of ERK1/2 activation reduces cell proliferation [31].

UV-A, UV-B and UV-C are the three wave bands of UV radiation, which is the main harmful component of sunlight. All UV radiations are genotoxic, and even if human lenses differ from those of rats or mice, components of UV light are capable of reaching the retina, as indicated by a structural study of rat retinas exposed to UV; notably, all of UV-A, UV-B, and UV-C were found to reach and affect the function of the retina [32]. In the present study, we observed that the stable HBx-transfected ARPE19 cells were more sensitive to UV-A and UV-B-induced damage at 1–5 J/cm^2^ than were the mock-transfected ARPE19 cells. It has been reported that UV-A and UV-B (1–2 J/cm^2^) irradiation can induce the apoptotic cell death of ARPE19 through severe nuclear and mitochondrial DNA damage. UV-B is more responsible for the DNA damage rather than UV-A irradiation. UV-B-induced DNA damage also results in the formation of pyrimidine dimers, whereas UVA-induced oxidative stress can also induce cellular damage of ARPE19 cells [33]. Interestingly, UV-A (20 J/cm^2^)-induced ARPE19 cell death can be rescued by the treatment of resveratrol and (−)-epigallocatechin gallate through the suppression of UV-A-induced MAPK and COX2 activation [34, 35].

In our in vitro study, cells expressing HBx were found to be similar to control cells regarding morphology and growth rate (Fig. 2). Moreover, HBx-expressing HepG2 cells were determined to exhibit increased sensitivity to apoptosis following UV irradiation [25]. Upon UV irradiation, the interaction of the HBx protein with DNA-binding protein (DDB) 1 prevents the degradation and improves the stabilization of the HBx protein [36], while further increasing cell death. Another DDB, DDB2, was revealed to enhance the translocation of HBx into the nucleus [37]. Both DDB1 and DDB2 are also responsible for DNA repair [38], although the DNA repair capacity is reduced when binding to the HBx protein [39].

By using microarray analysis, we further demonstrated that several cellular pathways are involved in the UV-induced cell death of HBx-expressing cells. Compared with the mock-transfected ARPE19 cells, the alterations of gene expression profiles in the HBx-expressing cells were classified into a total of 18 signaling pathways. Among them, the p53, TNF, and cancer-related pathways overlapped both pre- and post-UV irradiation. Notably, several metabolic processes were particularly altered in the HBx-expressing ARPE19 cells before UV irradiation, namely prostaglandin metabolism, cytokine–cytokine receptor interaction, ECM–receptor interaction, steroidogenesis, and the PI3K-Akt signaling pathway; in addition, BER, NER, MMR, HR, DNA replication, cell cycle regulation, and circadian rhythms were altered after UV irradiation (Table 4 and Fig. 5a). These data suggest that the processes between HBV infection and MD development are different, but share some pathogenic pathways. It has been known that UV induces different types of DNA damages, including cyclobutane-pyrimidine dimers (CPDs), 6-4 photoproducts (6-4PPs), as well as DNA strand breaks, which can be repaired by particular DNA repair pathways [40]. Notably, our results revealed that almost all genes in these UV-induced DNA repair pathways including BER, NER, MMR, and HR, were significantly inhibited in the HBx-transfected ARPE19 cells comparing with mock-transfected cells (Fig. 5b), suggesting that HBx protein sensitizes retinal epithelial cells to UV irradiation might be through down-regulation of multiple DNA repair pathways.

Our GO enrichment analysis results reveal that the DE genes were predominantly involved in molecular functions, biological processes, and cellular components. Notably, the most significantly affected genes involved in protein binding (Table 5A), DNA replication/cell cycle regulation (Table 5B), and the nucleosome/cytoplasm/nucleus (Table 5C) were particularly involved in HBx-expressing retinal pigment epithelial cells after UV irradiation, which might have contributed to the development of MD in the patients with HBV infection.

Notably, it has been shown that blue light (460 nm) irradiation can significantly induce apoptosis of ARPE19 cells through Bcl-2/BAX pathway [41]. Furthermore, the blue light irradiation also significantly induces the accumulation of reactive oxygen species and the mitochondrial dysfunction [3]. From our result, we further extend the notion that HBx may also sensitize the APRE-19 to blue light irradiation-induced cell death at 0.2–0.9 J/cm^2^ (Fig. 6), suggesting that HBV infection increases the sensitivity of retinal epithelial cells to both UV and blue light, thereby enhancing the risk of MD.

## Conclusion

We demonstrated that HBV infection raises the susceptibility to MD as well as to light damage from UV and short-wavelength visible light. We provided several lines of in vitro evidence to demonstrate that distinct signaling pathways are involved in HBx-expressing ARPE19 cells before and after UV irradiation. Our findings offer new understanding for the development of preventive strategies to address the mechanisms triggering HBV infection-associated MD in the future.

## Additional file

**Additional file 1: Figure S1.** The characteristics of the LED lamp. (A) The wavelength was measured by a spectrometer, showing a peak at 470 nm (blue light). (B) The chromaticity diagram was determined by a luminance colorimeter, showing that the LED lamp belonged to blue light area (arrow indicated black dot), (C) The irradiance of the blue LED lamp was approximate 26 W/m^2^ measured by a solar power meter.

## Abbreviations

AMD: age-related macular degeneration
BER: base excision repair
CPDs: cyclobutane-pyrimidine dimers
6-4PPs: 6-4 photoproducts
DDB: DNA-binding protein
HBV: hepatitis B virus
HBx: X protein of HBV
HR: homologous recombination
LHID: Longitudinal Health Insurance Database
ICD-9-CM: International Classification of Diseases, Ninth Revision, Clinical Modification
MD: macular degeneration
MMR: mismatch repair
NHRI: National Health Research Institutes
NHIRD: National Health Insurance Research Database
NHI: National Health Insurance
NER: nucleotide excision repair
ORFs: open reading frames
RPE: retinal pigment epithelial
UV: ultraviolet
TNF: tumor necrosis factor
