# Supplementary figures and images for: HBV infection increases the risk of macular degeneration: the roles of HBx-mediated sensitization of retinal pigment epithelial cells to UV and blue light irradiation
Source: J Transl Med. 2018 Aug 10;16:221. doi: 10.1186/s12967-018-1594-4 (PMC6086029; doi:10.1186/s12967-018-1594-4)

Figure S1

A

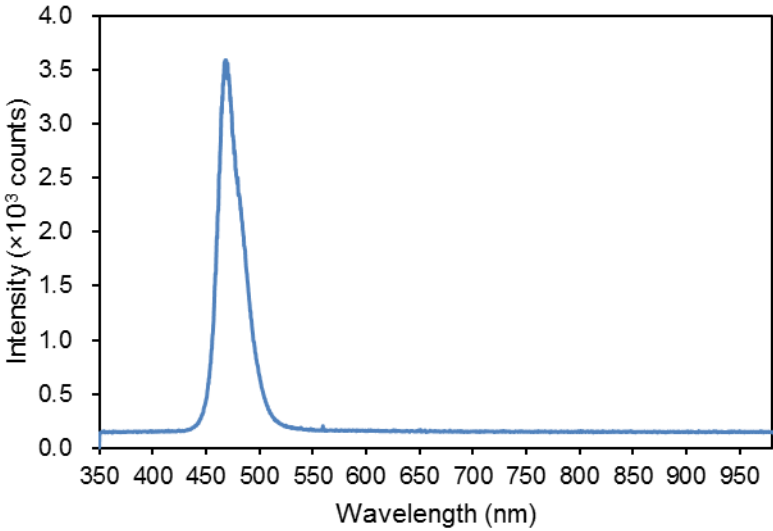

B

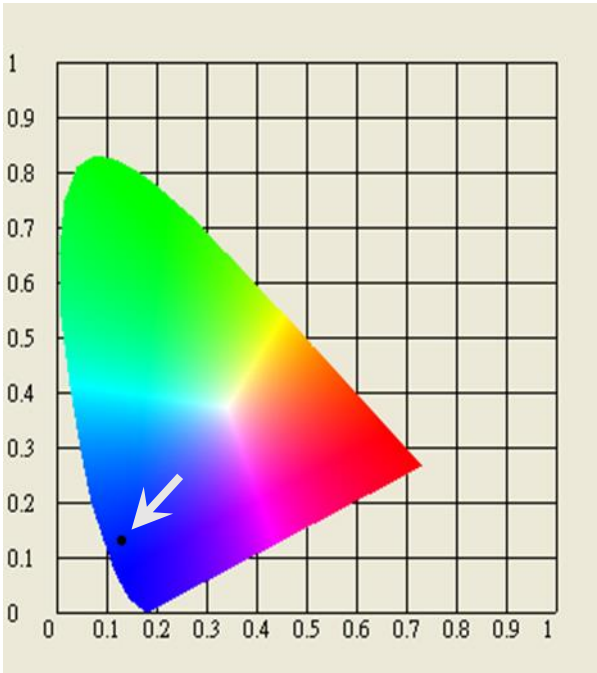

C

| Test No. | Value<br>(W/m <sup>2</sup> ) |
|----------|------------------------------|
| 1        | 25.7                         |
| 2        | 26                           |
| 3        | 26.1                         |
| 4        | 26.1                         |
| 5        | 26.1                         |
| 6        | 26.3                         |
| 7        | 26.3                         |
| 8        | 26.5                         |
| 9        | 26.5                         |
| 10       | 26.6                         |
| 11       | 26.6                         |
| 12       | 26.6                         |
| 13       | 26.6                         |
| Average  | 26.3                         |
| SD       | 0.29                         |
| CV       | 1.10%                        |

Supplement: Supplementary file 1 — Additional file 1: Figure S1. The characteristics of the LED lamp. (A) The wavelength was measured by a spectrometer, showing a peak at 470 nm (blue light). (B) The chromaticity diagram was determined by a luminance colorimeter, showing that the LED lamp belonged to blue light area (arrow indicated black dot), (C) The irradiance of the blue LED lamp was approximate 26 W/m2 measured by a solar power meter. [file 12967_2018_1594_MOESM1_ESM.pdf]
